# Why does diabetic retinopathy happen, and how can we stop it?

**Published:** 2011-09

**Authors:** Zoe Ockrim

**Affiliations:** Medical Retinal Fellow, Moorfields Eye Hospital, City Road, London, EC1V 9PD

**Figure:**
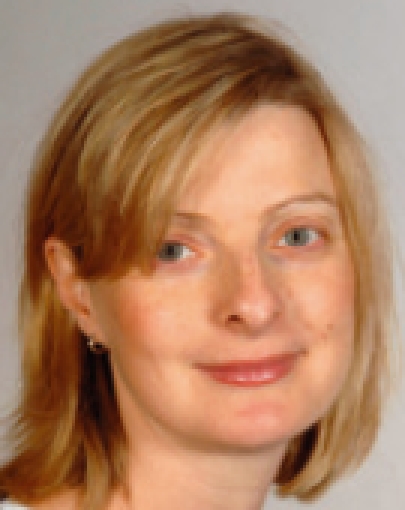


Diabetic retinopathy (DR) is a complication of diabetes. We can prevent DR both by preventing diabetes (primary prevention) and by improving the management of diabetes to slow down the onset, and reduce the severity, of DR (secondary prevention).

## Primary prevention

There is little that can be done to prevent type 1 diabetes. Its cause is uncertain and there is no evidence that any intervention can prevent it.

The vast majority of the 300 million people with diabetes have type 2 diabetes, which is often preventable. There is good evidence that lifestyle changes, such as losing weight, increasing physical activity, and eating more fruit and vegetables can lead to a significant reduction in the incidence of type 2 diabetes.

As diabetes is a cause of visual impairment, we should work with existing public health programmes and also ensure that diabetes is included in our eye care programmes. Eye care workers should take every opportunity to reinforce public health messages about avoiding obesity and taking regular exercise, and advise patients about weight loss and diet where possible. In addition, our specialist input might be valuable to the public health campaign, as avoiding blindness could be a powerful motivator for people to change their lifestyle for the better.

## Secondary prevention

### Optimal blood sugar control

Good blood sugar (glycaemic) control can reduce the risk of retinopathy in anyone with diabetes.

**Type 1 diabetes**. The Diabetes Control and Complications Trial followed two groups of people with type 1 diabetes, one that was intensively treated to control blood sugar and one that was managed in the usual way. After nine years they found a 26% reduction in the risk of development and progression of DR in the intensively treated group.

**Figure:**
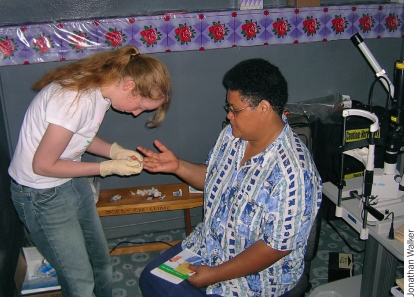
A volunteer checks the blood sugar of a patient in an eye clinic. FIJI

**Type 2 diabetes**. The United Kingdom Prospective Diabetes Study (UKPDS), published in 1997, showed that tight blood sugar control reduced the risk of progression of retinopathy and the need for laser treatment in people with type 2 diabetes. This study showed a 16% reduction in the risk of legal blindness in the intensively treated group compared to usual management at ten years.

In practice, however, perfect blood sugar control is unattainable in patients with type 1 diabetes because of the risk of unpredictable hypoglycaemia. With type 2 diabetes, most patients do not achieve tight control and if they do it tends to deteriorate with time.

**‘Avoiding blindness could be a powerful motivator for people to change their lifestyle for the better’**

### Control of blood pressure

In patients with high blood pressure (hypertension), control of blood pressure can reduce the risk of developing DR. The UKPDS randomised hypertensive patients with diabetes to two different groups:

- tight blood pressure control (<150/85 mmHg) using predominantly a beta-blocker or an angiotensin converting enzyme (ACE) inhibitor with the addition of other agents if required
- less tight blood pressure control (< 180/105 mmHg) without the use of beta-blockers or ACE inhibitors.

After seven years, there was a 35% reduction in the progression of DR in the tight blood pressure control group. At nine years, the tight blood pressure control group showed a 47% reduction in the risk of moderate visual loss and a 35% reduction in the need for laser treatment. The study found no benefit of the ACE inhibitor (captopril) over the beta-blocker (atenolol).

Several large studies have looked at the effect of individual ACE inhibitors in patients with diabetes. However, the effect on DR has been, at best, a secondary outcome measure and there is no clear evidence that one method of lowering blood pressure is superior to another in terms of its effectiveness in slowing down the progression of DR.

The Diabetic Retinopathy Candesartan Trials (DIRECT) were large randomised trials designed to assess whether reducing blood pressure in diabetic patients who did not have hypertension lowered the incidence/ progression of DR. The trials showed that there was no effect on either the incidence or progression of DR.

At present, therapies for established DR reduce the progression of the disease and stabilise visual acuity. Only rarely do these therapies lead to improved vision. Treating DR can be expensive. Every patient also requires monthly follow-up, which greatly increases the number of clinic visits.

The best and most affordable care we can provide for people with diabetes is secondary prevention to reduce the incidence of DR by means of good control of blood sugar and blood pressure. This can only be achieved if there is collaboration with physicians and good communication between eye health workers and patients. These measures will not only decrease the incidence and progression of DR, but also the other complications of diabetes, and they will be beneficial for every diabetes patient.

